# High prevalence of buccal ulcerations in largemouth bass, *Micropterus salmoides* (Centrarchidae) from Michigan inland lakes associated with *Myzobdella lugubris* Leidy 1851 (Annelida: Hirudinea)

**DOI:** 10.1051/parasite/2011181079

**Published:** 2011-02-15

**Authors:** M. Faisal, C. Schulz, A. Eissa, G. Whelan

**Affiliations:** 1 Department of Pathobiology and Diagnostic Investigation, College of Veterinary Medicine, Michigan State University East Lansing MI USA; 2 Department of Fisheries and Wildlife, College of Agriculture and Natural Resources, Michigan State University East Lansing MI USA; 3 Department of Fish Diseases and Management, Faculty of Veterinary Medicine, Cairo University Egypt; 4 Fisheries Division, Michigan Department of Natural Resources Lansing MI USA

**Keywords:** *Myzobdella lugubris*, leech, *Micropterus salmoides*, buccal ulcerations, *Myzobdella lugubris*, sangsue, *Micropterus salmoides*, ulcérations buccales

## Abstract

Widespread mouth ulcerations were observed in largemouth bass collected from eight inland lakes in the Lower Peninsula of Michigan during the summer months of 2002 and 2003. These ulcerations were associated with, and most likely caused by, leech parasitism. Through the use of morphological dichotomous keys, it was determined that all leeches collected are of one species: *Myzobdella lugubris*. Among the eight lakes examined, Lake Orion and Devils Lake had the highest prevalence of leech parasitism (34% and 29%, respectively) and mouth ulcerations (53% and 68%, respectively). Statistical analyses demonstrated that leech and ulcer prevalence varied significantly from one lake to the other. Additionally, it was determined that the relationship between the prevalence of ulcers and the prevalence of leech attachment is significant, indicating that leech parasitism is most likely the cause of ulceration. The ulcers exhibited deep hemorrhagic centers and raised irregular edges. Affected areas lost their epithelial lining and submucosa, with masses of bacteria colonizing the damaged tissues. Since largemouth bass is a popular global sportfish and critical to the food web of inland lakes, there are concerns that the presence of leeches, damaged buccal mucosa, and general unsightliness may negatively affect this important sportfishery.

## Abstract (fr)

La prévalence des ulcérations buccales du black-bass à grande bouche a été déterminée dans huit lacs intérieurs de la péninsule inférieure du Michigan pendant les mois d’été de 2002 et de 2003. Ces ulcérations étaient associées à un parasitisme par des sangsues. Par l’utilisation de clés dichotomiques morphologiques, toutes les sangsues récoltées ont été identifiées comme étant de l’espèce *Myzobdella lugubris*. C’est dans les lacs Orion et Devils que les plus hautes prévalences du parasitisme (34 % et 29 %) et des ulcérations buccales (53 % et 68 %) ont été observées. Les prévalences des sangsues et des ulcères buccaux variaient significativement d’un lac à l’autre. De plus, les prévalences des lésions buccales et du parasitisme étaient significativement liées, suggérant ainsi le rôle de la présence de sangsues dans la survenue des lésions buccales. Les ulcères se présentaient sous la forme d’une lésion centrale profonde et hémorragique avec des bords surélevés. L’épithélium et la sous-muqueuse des zones affectées avaient disparu et les tissus endommagés étaient colonisés par de nombreuses bactéries. Le black-bass à grande bouche est un poisson d’intérêt en pêche récréative, et il a un rôle majeur dans le réseau trophique des lacs intérieurs du Michigan. La présence des sangsues et de lésions buccales disgracieuses pourraient affecter négativement l’image et la santé de ce poisson prisé par les pêcheurs.

## Introduction

The largemouth bass (*Micropterus salmoides*) is an important species in the food web of inland lakes in the Laurentian Great Lakes basin (Bettoli *et al*., 1992). Due to its key predator-prey relationship with other aquatic organisms, largemouth bass plays a central role in the trophic cascades in the Great Lakes environment. In the state of Michigan, one-half million anglers predominately fish for largemouth bass, representing about 30% of all state licensed anglers (State of Michigan, 2004). Therefore, there is a need to better understand disease conditions, including parasitism, which can have an impact on this popular recreational fishery.

Leeches are known to feed upon a variety of terrestrial and aquatic animals, including fish (Sawyer, 1986). Leeches can be semi-permanent, attaching to only one host for the greater part of their adult life, or their attachment can be intermittent, attaching to several hosts throughout their lifetime (Sawyer, 1986). Adult leeches generally detach themselves from their fish hosts to deposit egg cocoons, from which juvenile leeches emerge after a few months to find their own fish hosts on which to feed, and grow into adults (Sawyer, 1986). The piscicolid leech *Myzobdella lugubris* is an intermittent blood-sucking parasite that parasitizes on a number of freshwater and estuarine fish species, including the largemouth bass, channel catfish (*Ictalurus punctatus*), white catfish (*I. catus*), mottled sculpin (*Cottus bairdi*), and burbot (*Lota lota*) (Daniels & Sawyer, 1975; Sawyer *et al*., 1975; Amin, 1981; Schramm *et al*., 1981; Muzzall *et al*., 1987; Morrison & Fox, 1993; Choudhury *et al*., 2004; Schulz *et al*., 2011). Interestingly, the life cycle of *M. lugubris* can involve a crustacean host on which this piscicolid leech deposits its egg cocoons, such as the blue crab, *Callinectes sapidus* (Daniels & Sawyer, 1975).

The preferred site of attachment for *M. lugubris* is primarily on the fin soft rays, causing erosion and epithelial hyperplasia (Dechtiar, 1972; Schulz *et al*., 2011). However, Noga *et al*. (1990) reported the presence of both ulcerations and attached *M. lugubris* in the mouth of four adult largemouth bass from Currituck Sound, North Carolina, though the relationship between the leech presence and the mouth ulcerations was never established. Herein, we report on widespread mouth ulcerations in adult largemouth bass caught from eight inland lakes of Michigan with a significant association with the presence of *M. lugubris*.

## Materials and Methods

### Fish and sampling sites

**Fig 1.**
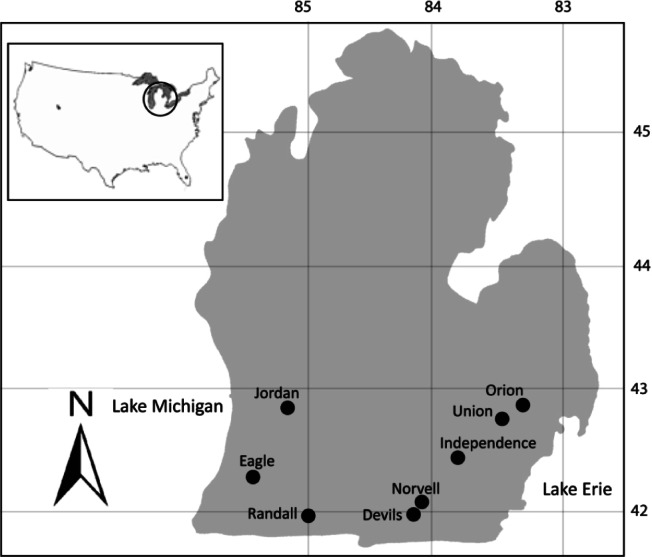
Largemouth bass (*Micropterus salmoides*) were collected from eight collection sites in the Lower Peninsula of Michigan between July 2002 and September 2003.

A total of 326 adult largemouth bass, *Micropterus salmoides*, with a mean total length of 30.2 cm ± 5.1 cm and mean total weight of 399.9 g ± 279.3 g were collected in summer months from eight inland lakes in Michigan’s Lower Peninsula between July 2002 and September 2003. The lakes sampled were Jordan Lake, Randall Lake, Norvell Lake, Devils Lake, Eagle Lake, Lake Orion, Union Lake, and Independence Lake (Fig. 1, Table I). Fish were collected primarily by electro-fishing, hook and line angling, and trap nets by biologists from the Michigan Department of Natural Resources. Fish were transported alive in tanks with aerators to the Aquatic Animal Health Laboratory at Michigan State University, East Lansing, Michigan. The number of fish samples from each lake is given in Table I.

**Table I.** Prevalence of mouth ulcerations and attached *Myzobdella lugubris* in largemouth bass (*Micropterus salmoides*) sampled from eight inland lakes in Michigan.

Lake	Latitude	Longitude	Number of fish examined	Number of fish with mouth ulcerations	Prevalence of mouth ulceration (%)	Number of fish with attached *M. lugubris* in the buccal mucosa	Prevalence of *M. lugubris* infection (%)
Devils Lake	41°59’01”N	84°17’13”W	28	19	68	8	29
Eagle Lake	42°10’12”N	85°58’32”W	48	11	23	11	23
Independence Lake	42°24’21”N	83°48’10”W	34	12	35	6	18
Jordan Lake	42°46’12”N	85°08’27”W	59	23	39	13	22
Norvell Lake	42°09’03”N	84°12’12”W	34	15	44	7	21
Lake Orion	42°46’56”N	83°15’01”W	32	17	53	11	34
Randall Lake	41°58’24”N	85°01’53”W	60	8	13	8	13
Union Lake	42°36’24”N	83°26’05”W	31	0	0	0	0

Total			326	105	32	64	20

### Laboratory and sample processing

Fish were anesthetized with 250 mg/liter Tricaine Methanesulfonate (MS-222, Argent Chemicals, Redmond, WA) and their buccal cavity thoroughly examined for the presence of ulcers and leeches. Leeches, when present, were collected, their numbers/fish recorded, and kept separately in vials containing saline (0.6% NaCl solution). Leeches were allowed to relax using menthol crystals (5-methyl-2-[1-methylethyl] cyclohexanol, Sigma-Aldrich Chemical Co., St. Louis, MO) added to a Petri dish of water and were then compressed gently with a glass slide (Pritchard and Kruse 1982). They were then fixed in an alcoholformalin- acetic acid (AFA) fixative for 24 hours and stored in 70% ethanol at room temperature until stained. Fixed leeches passed through descending strengths of ethanol (70%, 50%, and 35%), a single stage of distilled water, and were then stained with Mayer’s hematoxylin stain (Sigma), each immersion lasting 15-30 min, depending on the size of the leech. Once the leech was stained, it was dehydrated through ascending stages of ethanol immersion (85%, 95%, and 100%), lasting 15-30 min, and lastly, cleared by xylene (J.T. Baker, Phillipsburg, NJ). Mounted preparations from representative leeches were made and used for species identification (Pritchard & Kruse, 1982). Taxonomic identification of leeches followed the schemes of Sawyer *et al*. (1975), Khan & Meyer (1976), Barta (1991), Apakupakul *et al*. (1999), and Hoffman (1999).

### Statistical analyses

A one-way analysis of variance (ANOVA) was conducted to determine the significance of mouth ulcerations and leech prevalence. Additionally, Tukeyadjusted multiple comparisons were used to compare the prevalences of mouth ulcerations and leeches among lakes. A *P*-value less than 0.05 was considered significant. Calculations were performed using the SAS version 9.2 software (SAS Institute, Inc., Cary, North Carolina).

## Results

Examining the buccal cavity of largemouth bass revealed the presence of leeches crawling freely, attached to buccal mucosa, or buried in furrows within the mucosa. The overall prevalence of leeches in largemouth bass’ buccal cavity in the eight lakes combined was relatively high (20%, 64 out of 326 fish examined). Most fish harbored more than three leeches, but as many as six or seven leeches were common in fish from Lake Orion and Devils Lake. There were significant differences in leech prevalence among lakes with Lake Orion and Devils Lake being the highest at 34% and 29%, respectively (Table I). On the contrary, no leeches were attached to the buccal mucosa in fish from Union Lake.

**Fig 2.**
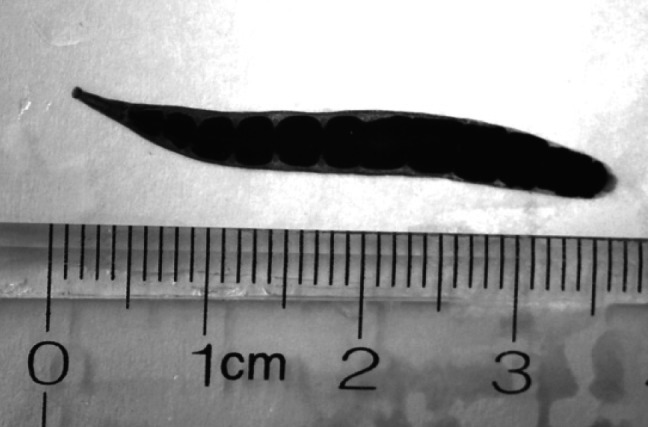
A specimen of *Myzobdella lugubris* compressed and stained with Mayer’s hematoxylin.

All leeches attached to the buccal mucosa of largemouth bass shared the same morphological criteria. Leech bodies were grayish to dark red, often contained scattered pigment cells, and measured 24-36 mm in length when relaxed (Fig. 2). All leeches exhibited the cylindrical body morphology; divided at the 13th segment into a narrow anterior and a wider posterior region; had one pair of eyes; and a concave caudal sucker.

**Fig 3.**
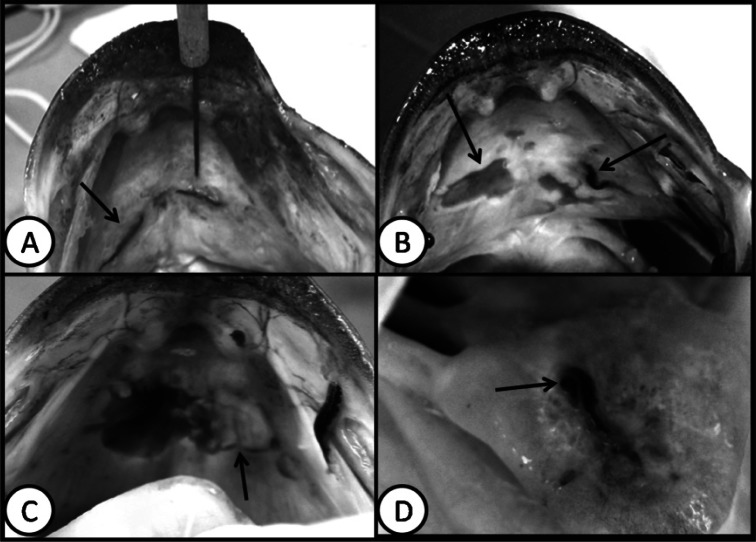
*Myzobdella lugubris*. (A) burrowing into the buccal mucosa of a largemouth bass (arrow) from Orion Lake; (B) ulcerations and leeches (arrows) in the roof of the mouth of a largemouth bass from Orion Lake; (C) severe ulceration associated with a leech infestation in the roof of the mouth of a largemouth bass from Devils Lake with suppurative inflammation and subsequent pus formation (arrow); and (D) *M. lugubris* (arrow) attached to the tongue of a largemouth bass from Devils Lake.

Clinical examination revealed the presence of widespread ulcerations of the buccal mucosa at the roof of the mouth as well as on the tongue mucosa. The prevalence of buccal ulcerations was relatively high since it was found in 105 out of 326 largemouth bass examined (32%). In several fish, leeches were found embedded in the buccal mucosa, particularly at the roof of the mouth (Fig. 3A). Even though leeches were within the mucosa, they were in continuous movement, a matter that caused detachment of the mucosal layer. Most of the ulcers exhibited deeper hemorrhagic centers with inflamed irregular edges (Fig. 3B). The size of ulcers varied from minute to relatively large. In advanced cases, ulceration covered a considerable portion of the buccal mucosa, often with yellowish-white material (Fig. 3C). In some instances, ulcers were found on the tongue with larger hemorrhagic areas surrounding the leech attachment sites (Fig. 3D).

Similar to leech prevalence, buccal ulceration prevalence was highest in Lake Orion (53%) and Devils Lake (68%) and did not exist in the leech-free Union Lake. Moreover, ulcer prevalence varied significantly among lakes. For example, the prevalence of ulcers in fish from Devils Lake was significantly higher than Eagle, Independence, Randall, and Union lakes (*P* < 0.01). The difference in ulceration prevalence between Randall Lake and Jordan and Norvell lakes was statistically significant (*P* < 0.05). Additionally, the prevalence of ulcers in fish from Lake Orion was greater than the prevalence of ulcers in fish from Randall, Union, Eagle, and Independence lakes, and was statistically significant (*P* < 0.05). When the individual fish data of the eight lakes was combined, statistical analysis revealed that the interaction between the prevalence of ulcers and the prevalence of leeches was statistically significant (*P* < 0.05).

## Discussion

Morphological criteria indicated that the leeches collected in this study were all *Myzobdella lugubris* Leidy 1851 (Sawyer *et al*., 1975; Khan & Meyer, 1976; Barta, 1991; Hoffman, 1999). It was surprising that *M. lugubris* was noticed only in the buccal cavity, since it is known also to attach to skin (Miller & Olson, 1973; Muzzall *et al*., 1987; Morrison & Fox, 1993; Choudhury *et al*., 2004), fins (Daniels & Sawyer, 1975; Schramm *et al*., 1981; Muzzall *et al*., 1987; Morrison & Fox, 1993), and isthmus (Schramm *et al*., 1981; Morrison & Fox, 1993). Moreover, statistical analyses revealed that *M. lugubris* prevalence is significantly related to the buccal ulceration, suggesting that the leech feeding habits and ability to burrow into the mucosa are most likely the cause of these ulcers. Indeed, most of the recent ulcers observed took the size and shape of the *M. lugubris* contour. The ulceration is probably exacerbated by the intermittent nature of *M. lugubris* parasitism, which is associated with continuous attachment, detachment, and movement into other parts of the mucosa. This study and that of Noga *et al*. (1990) in the largemouth bass are the only reports of *M. lugubris* having a tendency toward buccal mucosa.

*Myzobdella lugubris* produces two types of secretions that lyse host epithelium and aid in the attachment to host surfaces (Michel & Devillez, 1978; Appy & Cone, 1982). The tissue damage caused by this leech compromises the integrity of bass buccal mucosa and allows for potential invasion by pathogenic bacteria and viruses. In this context, recent studies from the authors’ laboratory have demonstrated that *M. lugubris* carries the Viral Hemorrhagic Septicemia Virus (Faisal & Schulz, 2009), a disease that has devastated important fish stocks in the Laurentian Great Lakes; and *Flavobacterium psychrophilum*, the causative agent of both Bacterial Coldwater Disease and Rainbow Trout Fry Syndrome, which can cause high mortalities in salmonids (Schulz & Faisal, 2010). Other species of leeches are well known for functioning as vehicles for transmission of infectious agents in fish (Dombrowski, 1952; Ahne, 1985; Cusack & Cone, 1986; Kruse *et al*., 1989; Jones & Woo, 1992; Zintl *et al*., 2000; Kikuchi *et al*., 2002; Hemmingsen *et al*., 2005; Kikuchi & Fukatsu, 2005). Whether or not *M. lugubris* transmits pathogens to the largemouth bass is currently unknown and deserves further investigation.

Prevalence of *M. lugubris* varied greatly from one lake to the other. Many biological, chemical, and physical components of each waterbody determine the presence and infection parameters of a certain parasite in a locale, including the presence and density of susceptible hosts, the prevailing temperature, the composition of the fish community, and the presence of other parasite species (Kennedy, 2009). Unfortunately, the information available on the hydrobiology of each of the lakes is not enough to allow identification of the factors that led to high leech prevalence in lakes Orion and Devils or its total absence in Union Lake. The presented data represents the most comprehensive account of *M. lugubris* prevalence in largemouth bass in Michigan’s inland lakes. The inland lakes from which largemouth bass samples were collected were never examined previously for leeches of largemouth bass. Therefore, the findings from this study are considered to be new geographical range extensions for *M. lugubris*.

### Declaration of New Geographic Range for *Myzobdella Lugubris* Leidy 1851

. Prevalence: varied from 0-34 (average 20%).
. Site of infection: buccal cavity.
. Type host: largemouth bass.
. Other reported hosts: *Acipenser brevirostrum*, *Acrocheilus alutaceus*, *Ambloplites rupestris*, *Ancylopsetta quadrocellata*, *Aplodinotus grunniens*, *Awaous guamensis*, *Callinectes sapidus*, *Carpiodes cyprinus, Catostomus macrocheilus*, *C. columbianus*, *Cottus bairdi*, *Eleotris sandwicensis*, *Esox lucius*, *Fundulus grandus*, *F. heteroclitus*, *F. majalis*, *F. similes*, *Ictalurus catus*, *I. melas*, *I. natalis*, *I. nebulosus*, *I. punctatus*, *Lepomis cyanellus*, *L. macrochirus*, *Lota lota*, *Micropterus dolomieu*, *M. salmoides*, *M. saxatilis*, *Moxostoma macrolepidotum*, *Mugil cephalus*, *Notemigonus crysoleucas*, *Notropis atherinoides*, *Palaemontetes pugio*, *Paralichthys lethostigma*, *Penaeus setiferus*, *Perca flavescens*, *Percina caprodes*, *Pomoxis annularis*, *P. nibromaculatus*, *Pterois volitans*, *Ptychocheilus oregonensis*, *Sander vitreus*, and *Sicyopterus stimpsoni*.
. New location(s) based on the present study: the following inland lakes in Michigan’s Lower Peninsula: Lakes Devil, Eagle, Independence, Jordan, Norvell, Orion, Randall, and Union.
. Other reported localities in North America: The U.S. states of Alabama, Arizona, California, Connecticut, Florida, Georgia, Hawaii, Kentucky, Louisiana, New Jersey, Maryland, Massachusetts, Michigan, Mississippi, North Carolina, South Carolina, Texas, Virginia, Washington, and West Virginia. It was also reported from the Canadian provinces of New Brunswick and Ontario.
. Representative publications: Appy & Dadswell, 1981; Becker & Dauble, 1979; Choudhury *et al*., 2004; Font & Tate, 1994; Klemm, 1972; Klemm, 1982; Klemm, 1991; Miller & Olson, 1973; Sawyer, 1972; and Sawyer *et al*., 1975.
. Specimen deposited: AMNH, in submittal process.
